# Attitudes Toward COVID-19 Vaccines in Chinese Adolescents

**DOI:** 10.3389/fmed.2021.691079

**Published:** 2021-07-07

**Authors:** Hong Cai, Wei Bai, Shou Liu, Huanzhong Liu, Xu Chen, Han Qi, Rui Liu, Teris Cheung, Zhaohui Su, Chee H. Ng, Yu-Tao Xiang

**Affiliations:** ^1^Unit of Psychiatry, Department of Public Health and Medicinal Administration & Institute of Translational Medicine, Faculty of Health Sciences, University of Macau, Taipa, China; ^2^Centre for Cognitive and Brain Sciences, University of Macau, Taipa, China; ^3^Institute of Advanced Studies in Humanities and Social Sciences, University of Macau, Taipa, China; ^4^Department of Public Health, Medical College, Qinghai University, Xining, China; ^5^Department of Psychiatry, Chaohu Hospital of Anhui Medical University, Hefei, China; ^6^The National Clinical Research Center for Mental Disorders & Beijing Key Laboratory of Mental Disorders, Beijing Anding Hospital & the Advanced Innovation Center for Human Brain Protection, Capital Medical University, School of Mental Health, Beijing, China; ^7^School of Nursing, Hong Kong Polytechnic University, Kowloon, China; ^8^Center on Smart and Connected Health Technologies, Mays Cancer Center, School of Nursing, UT Health San Antonio, San Antonio, TX, United States; ^9^Department of Psychiatry, The Melbourne Clinic and St Vincent's Hospital, University of Melbourne, Richmond, VIC, Australia

**Keywords:** COVID-19 vaccines, adolescents, attitude toward vaccines, efficacy and safety of COVID-19 vaccines, acceptance of COVID-19 vaccines

## Abstract

**Background:** As COVID-19 vaccination programs are being implemented widely, it is important to examine the attitudes of adolescents toward the COVID-19 vaccine and its uptake. The aim of this study was to examine the acceptance of and attitudes toward COVID-19 vaccines, and their associated factors among adolescents in China.

**Methods:** This was a cross-sectional, observational study conducted between November 27, 2020 and March 12, 2021 using snowball sampling method. Basic sociodemographic characteristics, health-related information, severity of depressive and anxiety symptoms, and attitudes and behavior toward COVID-19 vaccines were assessed.

**Results:** Overall, 1,057 adolescents participated in this study, yielding a response rate of 89.3%. There were 799 (75.59%) [95% Confidence Interval (CI) 73.00–78.18%] adolescents who would accept future COVID-19 vaccination. Binary logistic regression analysis revealed that adolescents who previously heard about COVID-19 vaccines (*P* = 0.001, odds ratio (OR) = 1.90, 95%CI:1.32–2.74), who thought that COVID-19 vaccines could protect them from COVID-19 infection (*P* = 0.002, OR = 2.93, 95%CI: 1.49–5.70), and those who encouraged their family members and friends to get vaccinated (*P* < 0.001, OR = 12.19, 95%CI: 6.78–21.92) and who believed that vaccines are safe (*P* = 0.012, OR = 3.94, 95%CI: 1.36–11.44) were more likely to accept future COVID-19 vaccination. In addition, younger adolescents (*P* = 0.003, OR = 0.93, 95%CI: 0.89–0.98) were more likely to accept future COVID-19 vaccines than older adolescents.

**Conclusions:** In conclusion, Chinese adolescents appeared to have positive attitudes toward COVID-19 vaccines. It is important to increase public confidence and knowledge regarding the efficacy and safety of COVID-19 vaccines to maximize the success of vaccination programs.

## Introduction

Coronavirus disease 2019 (COVID-19) caused by Severe Acute Respiratory Syndrome Coronavirus 2 (SARS-CoV-2) has been declared a pandemic on March 11, 2020 by the World Health Organization (WHO) (1). As of March 15, 2021, there were over 120 million COVID-19 cases and more than 1 million deaths caused by COVID-19 reported (2). Although China has proactively controlled the rapid transmission of COVID-19, sporadic cases of COVID-19 cases have persisted. Given the global impact of the COVID-19 pandemic on health and economy, effective infection control strategies to contain the virus transmission is the utmost priority among public health policymakers.

To date there are still no effective treatments for COVID-19 (3, 4). Vaccination is one of the most effective methods of controlling the COVID-19 pandemic by building herd immunity within the population. A multi-stage study comprising 9,542 residents in Wuhan, China found the proportion of participants who tested positive for COVID-19 pan-immunoglobulin was 6.92% (95%CI: 6.42–7.43), which suggests that vaccination would be necessary to achieve herd immunity and avoid future COVID-19 outbreaks (5). As of March 5, 2021, more than 200 vaccines were under development, of which more than 60 vaccines were in different stages of clinical trials, and at least seven vaccines have been rolled out (6). Many countries have started vaccination programs for certain subpopulations at high risk of infection (e.g., healthcare workers) in the first phase. In the next phase, other populations, such as healthy older adults, patients with chronic diseases, children and adolescents, will receive the COVID-19 vaccines (7). However, due to insufficient data on the efficacy and safety of vaccines in different subpopulations, the attitudes toward these COVID-19 vaccines have varied greatly (8).

According to a recent report examining the acceptability of COVID-19 vaccines among adults in the United States, 69% of participants were willing to accept the COVID-19 vaccines (9). Another survey conducted in China found that 91.3% of participants would accept the COVID-19 vaccines (10). In addition, a large-scale study comprising 13,426 adults in 19 countries found that 69% were likely to accept the COVID-19 vaccines (11). Commonly reported factors influencing vaccine acceptability and intention to be vaccinated included perceived benefit and efficacy of the COVID-19 vaccines (9, 10, 12). However, to date no study has examined the acceptability of COVID-19 vaccines and its associated factors among adolescents. Students are one of the most susceptible subpopulations to the COVID-19 pandemic because of their immature immune system and their insufficient capacity for self-protection from communicable diseases. Therefore, it is important to understand the attitudes toward COVID-19 vaccines in this subpopulation to achieve the public health goals of vaccination programs.

In this study we aimed to evaluate the acceptance of and attitudes toward COVID-19 vaccines and their associated factors among Chinese adolescents.

## Materials and Methods

### Study Design

This was a cross-sectional, observational study conducted between November 27, 2020 and March 12, 2021 using snowball sampling method through the collaborative research network of the National Clinical Research Center for Mental Disorders, China. To be eligible, participants should be secondary school students residing in China and able to understand the purpose and contents of the assessment. To avoid contagion during the COVID-19 pandemic, following previous studies (13, 14) data were collected online using the WeChat-based “Questionnaire Star” program. WeChat is a widely used smartphone-based social communication APP, with more than 1.2 billion active users in China. Following a previous study (15) using the WeChat-based “Questionnaire Star” program, all the questions in the assessments were set as “compulsorily answered” on an anonymous basis. Each assessment needed to be linked to a distinct IP address, and completed within a reasonable period based on a pilot study (the mean assessment time was 35 min in this study). All participants provided electronic written informed consent prior to participation in this study. Written consent from parents was sought for those participants aged under 18 years old. This study was approved by the Institutional Review Board (IRB) of Beijing Anding Hospital.

The sample size was calculated using the formula N = Zα^2^P (1 – P)/d^2^, in which α = 0.05 and Zα = 1.96, and the estimated acceptable margin of error for proportion d was 0.05. The acceptance rate of COVID-19 vaccines was estimated to be 69% based on a previous study (11). Assuming that 10% of those invited would refuse participation in this study, at least 361 participants had to be included.

### Measures

Basic sociodemographic characteristics and health related information were collected including age, gender, secondary school (junior/senior secondary school), residence (urban/rural), and perceived health status (bad/fair/good). Following a previous study on attitude about influenza vaccine (16), several standardized questions were added to measure attitudes and behaviors toward COVID-19 vaccines in this study, including: (1) “Do you worry about being infected with COVID-19?” (No/Fair/Very much); (2) “Have you heard of COVID-19 vaccines previously such as any information about COVID-19 vaccines, including both positive and negative news, vaccine development, safety and efficacy of vaccine via various channels (e.g., radio, television, telephone)?” (No/Yes); (3) “Do you think COVID-19 vaccines could protect you from COVID-19?” (No/Yes/No idea); (4) “How safe do you think COVID-19 vaccines are?” (Not safe with obvious side effects/No idea/Safe with no or minimal side effects); (5) “Would you encourage your family and friends to get vaccinated with the COVID-19 vaccine?” (No/Yes/No idea). An extra question was asked to examine participants' behavior toward COVID-19 vaccines: “Do you intend to be vaccinated against COVID-19 in the future?” (No/Yes).

Severity of the depressive symptoms was assessed using the Chinese version of the 9-item Patients Health Questionnaire (PHQ-9) (17, 18), with each item scoring from “0” (not at all) to “3” (nearly every day). The total scores ranged from 0 to 27. A higher score indicates more severe depressive symptoms. Severity of anxiety symptoms was measured using the Chinese version of the 7-item Generalized Anxiety Disorder Scale (GAD-7) (19, 20), which comprises 7 items with each scoring from 0 (not at all) to 3 (nearly every day) with a total score ranging from 0 to 21. A higher score indicates more severe anxiety symptoms.

### Statistical Analysis

Data analyses were performed using SPSS version 25.0 (SPSS Inc., Chicago, Illinois, USA). All continuous variables were checked for normal distributions using P-P plots. Chi-square tests, independent samples *t*-tests, and Mann-Whitney *U*-tests were used to compare socio-demographic and COVID-19 vaccine related variables between adolescents who would accept future COVID-19 vaccination and those who would not accept. In order to examine the independent correlates of acceptance of future COVID-19 vaccination, the variables that significantly differed in univariate analyses were entered as independent variables in binary logistic analysis with the “enter” method. Significant statistical difference was set at *P* < 0.05 (two-tailed).

## Results

**Figure 1:**
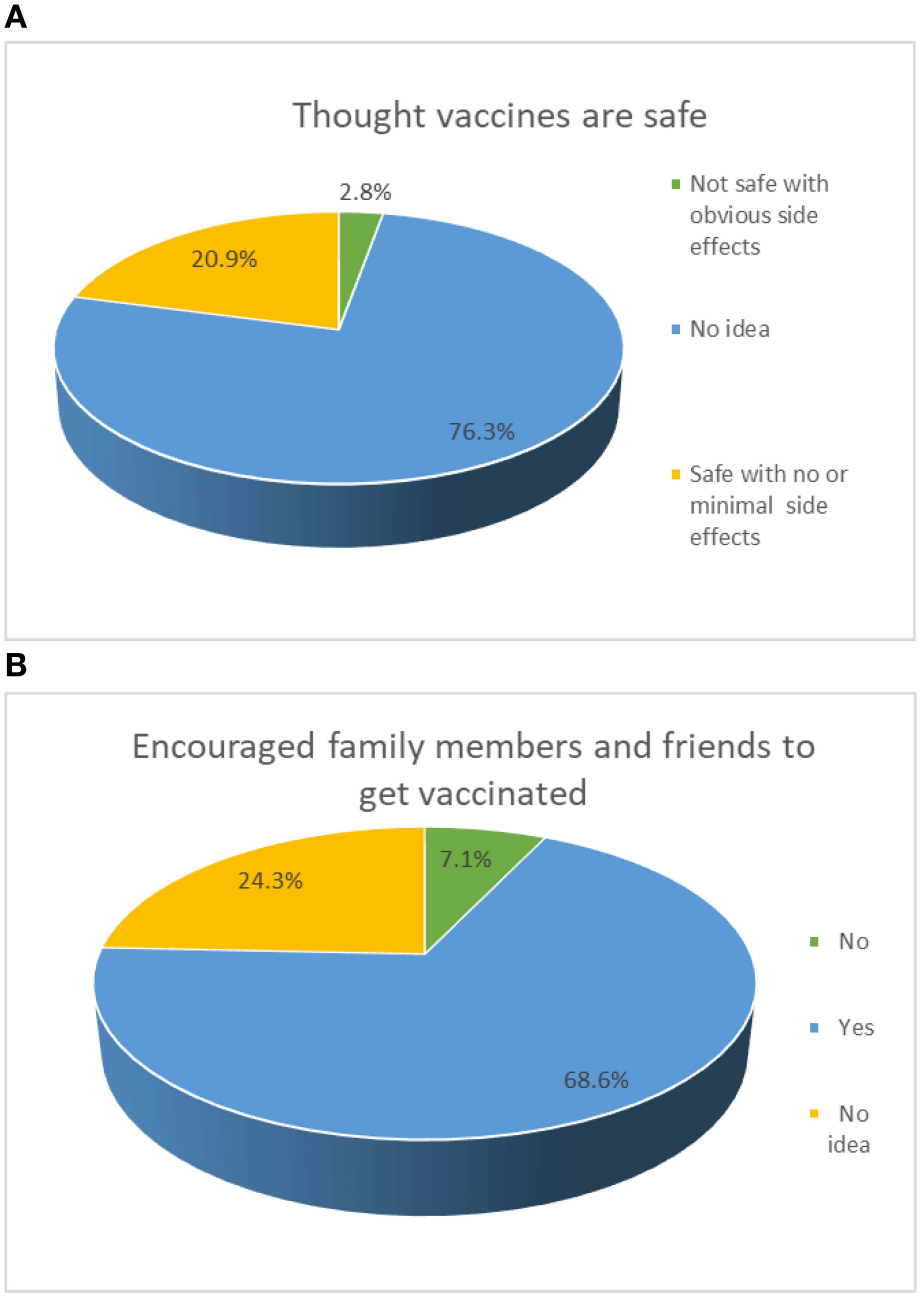
Confidence in COVID-19 vaccines.

Overall, 1,183 adolescents were invited to participate in the survey, and 1,057 completed the survey, yielding a response rate of 89.3%. There were 799 (75.59%, 95%CI: 73.00–78.18%) adolescents who would accept future COVID-19 vaccination. The mean age was 16.30 [standard deviation (SD): 3.61] years (range: 12–20 years) and 637 (60.3%) participants were female (Table 1). Of the participants, 76.3% (95%CI: 73.69–78.82%) believed that COVID-19 vaccines are safe with no or mild side effects, while 68.6% (95%CI: 65.79–71.39%) would encourage family and friends to get vaccinated with the COVID-19 vaccines (Figure 1).

**Table 1:** Demographic characteristics of participants.

**Variables**	**Total**		**Acceptance of COVID-19 vaccination**				**χ^2^**	***df***	***P***
	**(*****N*** **= 1,057)**		**Yes (*****N*** **= 799)**		**No (*****N*** **= 258)**				
	***N***	**%**	***N***	**%**	***N***	**%**			
Female gender	637	60.3	476	59.6	161	62.4	0.65	1	0.42
Urban residents	407	38.5	309	38.7	98	38.0	0.04	1	0.84
Junior secondary school	479	45.3	376	47.1	103	39.9	4.01	1	**0.045**
Perceived health status							2.02	2	0.37
Bad	7	0.7	4	0.5	3	1.2			
Fair	101	9.6	73	9.1	28	10.9			
Good	949	89.8	722	90.4	227	88.0			
Concern about COVID-19							3.24	2	0.20
No	17	1.6	10	1.3	7	2.7			
Fair	307	29.0	228	28.5	79	30.6			
Very much	733	69.3	561	70.2	172	66.7			
Worry about being infected with COVID-19							0.10	2	0.995
No	109	10.3	82	10.3	27	10.5			
Fair	631	59.7	477	59.7	154	59.7			
Very much	317	30.0	240	30.0	77	29.8			
Heard about COVID-19 vaccines previously	806	76.3	651	81.5	155	60.1	49.32	1	**<0.001**
Thought COVID-19 vaccines can provide protection							70.58	2	**<0.001**
No	73	6.9	36	4.5	37	14.3			
Yes	473	44.7	410	51.3	63	24.4			
No idea	511	48.3	353	44.2	158	61.2			
Encouraged family and friends to get vaccinated							234.99	2	**<0.001**
No	75	7.1	22	2.8	53	20.5			
Yes	725	68.6	644	80.6	81	31.4			
No idea	257	24.3	133	16.6	124	48.1			
Thought vaccines are safe							51.36	2	**<0.001**
Not safe with obvious side effects	30	2.8	10	1.3	20	7.8			
No idea	806	76.3	593	74.2	213	82.6			
Safe with no or minimal side effects	221	20.9	196	24.5	25	9.7			
	**Mean**	***SD***	**Mean**	***SD***	**Mean**	***SD***	**t/Z**	***df***	***P***
Age (years)	16.30	3.61	16.07	3.46	17.03	3.96	3.75	1,055	**0.001**
PHQ-9 total	3.98	5.44	3.81	5.31	4.50	5.81	−1.69	–^*^	0.09
GAD-7 total	2.67	4.40	2.50	4.30	3.18	4.66	−2.17	–^*^	**0.03**

*Bolded values, <0.05; M, mean; SD, standard deviation; COVID-19, Corona Virus Disease 2019; PHQ-9, 9-item Patient Health Questionnaire; GAD-7, 7-item Generalized Anxiety Disorder Scale*.

* *Mann-Whitney U-test*.

Table 1 shows the demographic characteristics and health related information of the participants and the comparisons between adolescents who would accept future COVID-19 vaccination and those who would not accept. There were significant differences between groups in terms of educational level, age, and anxiety symptoms, as well as the responses to the four questions about attitudes toward COVID-19 vaccines. No difference in depression was found between adolescents who accepted and those who did not accept future COVID-19 vaccination (*p* = 0.09).

Binary logistic regression analysis revealed that adolescents who heard about COVID-19 vaccines previously (*P* = 0.001, OR = 1.90, 95%CI:1.32–2.74), who thought COVID-19 vaccines can provide protection (*P* = 0.002, OR = 2.93, 95%CI: 1.49–5.70), who encouraged family members and friends to get vaccinated (*P* < 0.001, OR = 12.19, 95%CI: 6.78–21.92) and who thought vaccines are safe (*P* = 0.012, OR = 3.94, 95%CI: 1.36–11.44) were more likely to accept future COVID-19 vaccination (Table 2). In addition, younger adolescents (*P* = 0.003, OR = 0.93, 95%CI: 0.89–0.98) were more likely to accept future COVID-19 vaccination than older adolescents. No association between anxiety and COVID-19 vaccine acceptance (*p* = 0.27) was found (Table 2).

**Table 2:** Independent correlates of future COVID-19 vaccination acceptance by multiple logistic regression analysis.

**Variables**	**Multiple logistic regression analysis**		
	***P-*values**	**OR**	**95%CI**
Junior secondary schools	0.88	1.03	0.72–1.47
Heard about COVID-19 vaccines previously	**0.001**	**1.90**	**1.32–2.74**
**Thought COVID-19 vaccines can provide protection**			
No	–	1.0	–
Yes	**0.002**	**2.93**	**1.49–5.70**
No idea	**0.033**	**2.04**	**1.06–3.93**
**Encouraged family members and friends to get vaccinated**			
No	–	1.0	–
Yes	**<0.001**	**12.19**	**6.78–21.92**
No idea	**0.026**	**2.00**	**1.09–3.67**
**Thought vaccines are safe**			
Not safe with obvious side effects	–	1.0	–
No idea	**0.016**	**3.30**	**1.25–8.74**
Safe with no or minimal side effects	**0.012**	**3.94**	**1.36–11.44**
Age (years)	**0.003**	**0.93**	**0.89–0.98**
GAD-7 total	0.27	0.98	0.95–1.02

*Bolded values, <0.05; CI, confidential interval; OR, odds ratio; COVID-19, Corona Virus Disease 2019; GAD-7, 7-item Generalized Anxiety Disorder Scale*.

## Discussion

To the best of our knowledge, this was the first study that examined the attitudes of adolescents toward COVID-19 vaccines and their associated factors. Our findings were similar to other international findings that around 69–80% of adults would accept COVID-19 vaccination in countries such as England, Denmark, the US, Australia and France (9, 11, 21–24). In addition, no associations of COVID-19 vaccine acceptance with anxiety or depression were observed in this survey. However, our results were lower than the previous figures found among Chinese adults who had a COVID-19 vaccination acceptance rate of 91.3% (10). The main reasons for the lower vaccine acceptance rate in adolescents may include uncertainty about vaccine safety and efficacy, and inadequate knowledge about the potential benefits of vaccination among children and adolescents (21). In addition, insufficient clinical trial data, lack of knowledge about the vaccine and the COVID-19 may increase misconceptions about COVID-19 vaccines (25). Further, the possibility of pain and discomfort associated with vaccination could also lead to a lower acceptance rate of the COVID-19 vaccines in adolescents. In this study, we found that younger adolescents were more likely to accept COVID-19 vaccination than older adolescents, possibly because younger adolescents are more compliant with positive directions and had limited access to online negative information or misinformation about vaccines than older adolescents.

Adolescents who heard about COVID-19 vaccines previously were more likely to accept future COVID-19 vaccination, which reflects a better recognition of the efficacy of vaccines in controlling the pandemic. In response to the profound impact of the pandemic, China has taken rigorous public health interventions to control the spread of COVID-19 since the outbreak of the disease (26, 27). Chinese residents often hold strong beliefs about the efficacy of COVID-19 vaccination and such a positive attitude may explain why adolescents who heard about COVID-19 vaccines previously were more likely to accept COVID-19 vaccination. According to the health belief model, the perceived benefit from vaccination is likely to outweigh the risks (28–30). As of March 15, 2021, nearly 65 million people in China have been vaccinated against COVID-19 (6, 8), with no severe adverse reactions reported so far. Further, in China tertiary general hospitals are located close to the vaccination sites which could provide timely and professional interventions to identify and respond to any adverse reactions from the COVID-19 vaccines. All these factors could increase public confidence of the COVID-19 vaccination programs among residents including adolescents.

Public concern about vaccine safety has frequently been reported as a major obstacle to the rollout of vaccination programs, especially for newly introduced vaccines in clinical practice (28, 31, 32, 36). For example, an online survey at the start of the vaccination program in China revealed that only 54.8% of the participants had (“probably yes”) intent to be vaccinated against COVID-19 (12). Positively, China has since established a vaccine regulatory system, with relevant vaccine regulations and a quality control system. Therefore, these safeguards around the development and implementation of the COVID-19 vaccination programs have increased the overall vaccine acceptance in the community at large.

Adolescents who encouraged family members and friends to get vaccinated were more likely to accept future COVID-19 vaccination. This may be largely driven by their concern to protect their family members and friends from contracting COVID-19. If participants believe that their family members and friends are in the high-risk groups, they would encourage them to have the COVID-19 vaccination (33). Studies have shown that parents' values and attitudes can be influenced by their children (34, 35). For instance, in a study conducted in eastern Zambia, school-age children and adolescents are change agents who had a positive impact on their families' or friends' health behaviors such as the practice of water, sanitation, and personal hygiene (34).

The strengths of this study included the large sample size and the focus on the adolescent population covering many provinces in China. However, there are several limitations that should be addressed. First, due to the cross-sectional study design, the casual relationship between attitudes toward COVID-19 vaccines and other variables could not be established. Second, although the attitudes toward COVID-19 vaccines in adolescents may largely depend on their parents or guardians, we did not measure the attitudes of the parents or legal guardians of the participants. Hence, the study could not reflect the potential coverage of vaccines in adolescents in the future, similar to previous studies (21). Third, due to logistical reasons, participants were recruited by a snowball, rather than random sampling method, which may limit the generalizability of the findings. Fourth, there were several minor outbreaks caused by imported cases from overseas in January 2021 in Hebei, Heilongjiang, and Jilin provinces, which may have influenced the adolescents' attitudes toward COVID-19 vaccines to an uncertain extent. Lastly, the standardized questions on attitudes and behaviors toward COVID-19 vaccines used in this survey were based on a previous study on attitude about influenza vaccine (16). Hence, certain additional information, such as having no idea about the attitude toward COVID-19 vaccines, could not be included in the study.

In conclusion, Chinese adolescents appeared to have a relatively high rate of acceptance of COVID-19 vaccination. It is important to increase the public confidence and knowledge regarding the efficacy and safety of COVID-19 vaccines to maximize the success of vaccination programs.

## Data Availability Statement

The Institutional Review Board (IRB) of Beijing Anding Hospital that approved the study prohibits the authors from making the research data set publicly available. Readers and all interested researchers may contact Dr. Yu-Tao Xiang (Email address: xyutly@gmail.com) for details. Dr. Xiang could apply to the Institutional Review Board (IRB) of Beijing Anding Hospital for the release of the data.

## Ethics Statement

The studies involving human participants were reviewed and approved by the Institutional Review Board (IRB) of Beijing Anding Hospital. Written informed consent to participate in this study was provided by the participants' legal guardian/next of kin.

## Author Contributions

Y-TX and CN: study design. HC, WB, SL, HL, XC, HQ, and RL: collection, analyses, and interpretation of data. HC, CN, and Y-TX: drafting of the manuscript. TC and ZS: critical revision of the manuscript. All the authors approval of the final version for publication.

## Conflict of Interest

The authors declare that the research was conducted in the absence of any commercial or financial relationships that could be construed as a potential conflict of interest.
